# Assessment of hardening due to non-coherent precipitates in tungsten-rhenium alloys at the atomic scale

**DOI:** 10.1038/s41598-019-52521-x

**Published:** 2019-11-07

**Authors:** G. Bonny, A. Bakaev, D. Terentyev

**Affiliations:** SCK•CEN, Nuclear Materials Science Institute, Boeretang 200, B-2400 Mol, Belgium

**Keywords:** Mechanical properties, Metals and alloys

## Abstract

In metallurgical applications, precipitation strengthening is of great technological importance to engineer materials with the required strength. While precipitation hardening is essential for many applications involving operation at elevated temperatures, its subsequent embrittlement can be a showstopper for the overall performance of a component. In the nuclear industry, irradiation-induced/enhanced precipitation and the resulting embrittlement often limit the lifetime of components. In fusion applications, tungsten (W) based alloys are known to harden and embrittle as a result of irradiation-assisted transmutation to rhenium (Re) and its subsequent precipitation into non-coherent precipitates. Hence, a fundamental understanding of the interaction of dislocations with non-coherent precipitates is of great interest. In the present work, the interaction of dislocations with non-coherent Re-rich *σ*, *χ* and hcp phase precipitates embedded in a bcc W matrix is assessed. Large-scale atomistic simulations are performed to clarify the interaction mechanisms and derive the obstacle strength of the precipitates in the quasi-static limit. Thereby the impact of precipitate shape, size, interspacing and composition is assessed. Based on those results, an analytical model to predict precipitation hardening of *σ*, *χ* and hcp phase particles in bcc W is proposed and compared to available experimental data from mechanical tests on irradiated materials.

## Introduction

In metallurgy, high toughness and proof stress of structural materials and composites can be realized via various strengthening mechanisms, such as, solid solution strengthening, dislocation strengthening, phase transformations, etc.^1^. Amongst these mechanisms, precipitation strengthening is an important one. In this case, the strengthening is achieved by the obstruction of dislocation movement by secondary phase particles; strengthening of steels by carbides being one of the most famous examples. In many cases, the precipitated phase is an intermetallic that is not coherent with the host matrix. It is this class of precipitates, so-called non-coherent precipitates, that obstructs dislocation motion most effectively and causes the strongest hardening effect, resulting in the overall strengthening of the material.

For many technological applications, precipitation hardening is essential to improve the alloys’ strength, e.g., precipitation strengthening of Al-based alloys used in the aerospace industry, carbo-nitride precipitation to improve the high temperature strength of ferritic-martensitic steels, etc. However, the embrittlement associated to undesired precipitation and subsequent hardening can be detrimental to the performance of materials under extreme loading conditions. The so-called “475 °C embrittlement” in stainless steels, expressed through the formation of Cr rich α’ precipitates, is a well-known case of embrittlement of structural steels^2^. The formation of topologically close-packed phases in Re-containing super alloys is another expression of embrittlement caused by undesired precipitation^3^. These well-registered examples show that the precipitation of secondary phase particles must be controlled, and if they cannot be excluded, their consequences to the degradation of the mechanical properties should be understood.

The precipitation of non-coherent secondary phase particles in tungsten-rhenium alloys (W-Re) is a very important subject that is so far scarcely addressed. Tungsten is the material of choice for the plasma-facing components in the International Thermonuclear Experimental Reactor (ITER) and DEMOnstration Power Station (DEMO)^4,5^, where the strength and structural integrity play a critical role with respect to the durable and safe exploitation of the reactor. In recent years, different aspects of the degradation of tungsten under fusion operational conditions were considered to highlight the impact of: high temperature in-service recrystallization^6^, hardening induced by plasma components^7^, the evolution of radiation defects by direct *in-situ* ion exposure^8^ and others (see ref.^5^ for a recent review). The role of irradiation-induced precipitation so far remains underexplored, especially by computer simulation tools, even though the few experimental reports highlight the strong impact of Re precipitation on the degradation of the mechanical properties^9–11^.

The formation of the second phase Re-rich precipitates in W under fusion conditions occurs because of nuclear transmutation reactions (W → Re, Re → Os, etc.) coupled with irradiation-induced diffusion/segregation. Following the preliminary design assessment of DEMO, after the first 5 years of full power operation, a few percent (3–6%) of Re will form in W on both first wall and divertor components^12^. Although this concentration is well below the solubility limit, the irradiation-induced and –enhanced diffusion will cause the precipitation of Re, which was observed in fission irradiation experiments^9–11^. Researchers have reported the formation of non-coherent *σ* and *χ* phase particles, based on transmission electron microscopy (TEM) that evidently increases the flow stress of the material as revealed by micro-hardness tests^13–17^. The most recent atom probe tomography investigations highlight that some of the Re-rich precipitates formed under irradiation might not be resolvable in TEM. While these precipitates are rather small, their volumetric density is high (up to 2 × 10^24^ m^−3^)^9^. Thus, experimentalists call for a theoretical assessment of the interaction of nano-scale Re-rich precipitates with dislocations to progress our fundamental understanding of the performance of W under fusion operating conditions.

In this contribution, the interaction of a gliding dislocation with non-coherent *σ*, *χ* and hcp Re-rich precipitates embedded in a bcc W matrix is investigated. The spacing and dimensions of the precipitates are chosen in line with recent experimental data reported in^9^. Large-scale atomistic simulations are performed in the quasi-static limit to clarify full details of the interaction mechanisms at the atomic level and to obtain the obstacle strength of the precipitates. Thereby the impact of precipitate shape, size, interspacing and composition is parametrically assessed. The obtained results are used to derive an analytical model to extrapolate precipitation hardening for *σ*, *χ* and hcp phase particles of larger sizes in bcc W. The resulting model is compared to experimental data from Vickers micro-hardness tests.

## Methods

The interaction between an edge dislocation and precipitate was modelled using molecular statics (MS) implemented in the large scale atomic molecular massively parallel simulator (LAMMPS)^18^ in combination with the W-Re potential developed by Bonny *et al*.^19^. The W-Re potential is based on the EAM2 potential for W by Marinica *et al*.^20^, which was fitted with a focus on the description of radiation defects and dislocation lines^20,21^. In particular, it describes the screw dislocation core structure and its Peierls barrier consistent with density functional theory (DFT) data. The W-Re interactions were fitted to the experimental elastic constants of the bcc W-Re solid solution, the DFT computed elastic constants of the W-Re intermetallic *σ* phase and the DFT predicted screw dislocations core structure in W-Re alloys^22^. Therefore, a priori, this potential is an excellent choice for the present study.

The simulation box with a 1/2$\langle 111\rangle$ edge dislocation was generated following the description by Osetsky *et al*.^23^, with *x*, *y* and *z* axes oriented along the $[\bar{1}12]$, $[1\bar{1}1]$ and $[110]$ directions, respectively. Periodic boundary conditions were applied along the *x* and *y* axes. In the simulation box, a region sufficiently separated from the edge dislocation was then removed and replaced by a precipitate, i.e., bcc, *σ*, *χ*, or hcp phase. The replacement was executed such that the total number of atoms in the box was approximately conserved.

**Figure 1:**
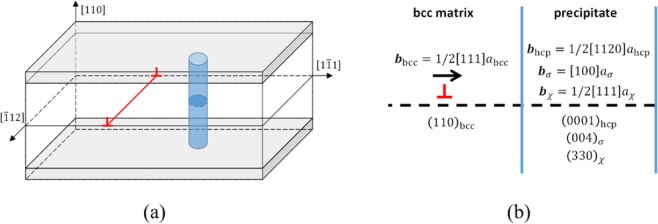
Illustration of (**a**) the box setup with cylindrical precipitate (similar for spherical); and (**b**) the orientation relationships between precipitate and matrix for close packed planes and the shortest primitive translation vector. Indicated in blue is the precipitate, in red the edge dislocation line and gray shaded the fixed regions that do not participate in the relaxation cycle.

As illustrated in Fig. 1, the simulation box was then separated in three regions along the *z*-axis: the top and bottom regions with a height of ~1 nm, where the atoms were kept fixed and the center region where the atoms were relaxed following the conjugate gradient method. After each relaxation cycle, the top region of atoms was rigidly displaced to realize a strain step of 10^−6^–10^−5^, depending on the specific configuration. This simulation scheme allowed stable glide of the edge dislocation in the (110) glide plane. After each strain step, the resolved shear stress was computed as the total force exerted on the bottom region of fixed atoms, *F*_*y*_, divided by the *xy* surface area. This box setup allows the study of the interaction of an edge dislocation with a precipitate under quasi-static conditions at zero Kelvin.

Both cylindrical and spherical precipitates were introduced, corresponding to needle shaped *χ* precipitates and equiaxed *σ* precipitates, respectively. The diameter of the spherical precipitates was varied in the range 3–5 nm, while the diameter for cylindrical precipitates was varied in the range 1–3 nm, both in the range of experimental observations^9,13–17^. The precipitate interspacing was taken in the range 10–40 nm, corresponding to precipitate number densities in the range 10^22^–10^24^ m^−3^, consistent with experiments^9,13–17^. For these parameters boxes with dimensions 55 × (10–40) × 38 nm^3^ corresponding to 1.3–5.1 × 10^6^ atoms were used.

The *σ*, *χ* and hcp phase precipitates were oriented such that their close packed planes coincide with the bcc close packed plane, i.e., the (110) edge dislocation glide plane and the shortest primitive translation vector parallel with the Burgers vector ***b*** = 1/2$\langle 111\rangle$, as schematically illustrated in Fig. 1b. To identify the close packed planes for the *σ*, *χ* and hcp phases, the procedure proposed by Kelly *et al*.^24^ was used, where the planar atomic density is estimated based on the product of the structure factor and interplanar spacing. Application of the latter procedure yields the (004), (330) and (0001) as close packed planes for the *σ*, *χ* and hcp phases, respectively. The primitive translation vectors for *σ*, *χ* and hcp phases are $\langle 100\rangle$, 1/2$\langle 111\rangle$ and 1/2$\langle 1120\rangle$, respectively.

The composition of the precipitates was varied in the range 50–75% for bcc, 50–75% Re for *σ*, 50–100% Re for *χ*, and 75–100% Re for hcp. It is noted that 100% Re for bcc and <75% Re for hcp were omitted as for these compositions the phases are not mechanically stable^19^. Both the *σ* and *χ* unit cells consist of five and four sub-lattices, respectively. Given the global Re concentration, the different sub lattices have different Re compositions. For both phases, the compositions for the sub-lattices reported in^22,25,26^ were used. The latter are based on thermodynamic computations consistent with experiments.

Visualizations, dislocation and adaptive common neighbor analyses were performed using the open visualization tool (OVITO)^27,28^.

## Results

### Coherent precipitates

In the following, the simulation results for bcc Re-rich coherent precipitates are presented. These results are taken as reference for the interaction mechanism and critical resolved shear stress, *τ*_*C*_. All performed simulations with different Re concentration, *C*_Re_, precipitate shape, diameter, *d*, and spacing, *L*, are summarized in the Supplementary Material. The *τ*_*C*_ is reported in units of $\frac{\mu b}{L^{\prime} }$ and can be interpreted as the obstacle strength, $\alpha =\frac{{\tau }_{C}L^{\prime} }{\mu b}$, with *μ* the shear modulus of the W host matrix (*μ* = 160 GPa), *b* the length of the Burgers vector (*b* = 0.272 nm) and *L*′ = *L* − *d* the free passage length between obstacles (precipitates).

**Figure 2:**
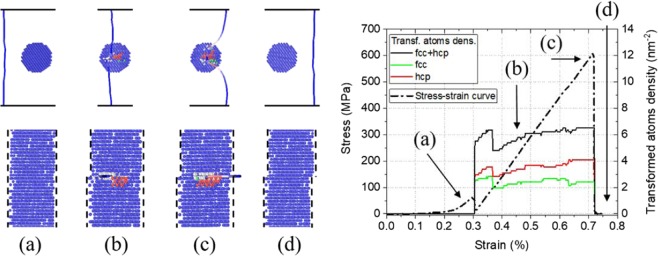
Schematic overview of the interaction between an edge dislocation and a cylindrical bcc 75% Re precipitate (left panel); and a representative stress-strain curve with superposed also the density of transformed atoms, *ρ*_tr_ (right panel). The dislocation line is color coded following its character: blue for pure edge character, red for pure screw character and white for mixed character. Only the Re atoms are visualized and color-coded following a common neighbor analysis: blue – bcc; green – fcc; red – hcp; grey – unidentified due to the dislocation core.

A representative stress-strain curve and schematic overview of the dislocation-precipitate interaction mechanism are shown in Fig. 2. Initially, a slight repulsion between the dislocation and precipitate is observed (a). Upon penetration of precipitate by the dislocation, locally, around the dislocation core, the bcc coordination of the atoms in the precipitate is changed from bcc into fcc and hcp coordination. The density of transformed atoms, *ρ*_tr_, defined as the number of transformed atoms normalized over the shearable area of the precipitate, *πd*^2^/4, is superposed in Fig. 2.

The transformed atoms pin the dislocation inside the precipitate, leading to a curvature in the dislocation. As a result, the stress increases linearly up to the critical resolved shear stress (c). During the pinning and corresponding stress accumulation, *ρ*_tr_ remains quasi constant (see Fig. 2). Upon unpinning, the precipitate is sheared, and all atoms return to a bcc coordination (d). It is noted that during the interaction, *ρ*_tr_ is slightly higher for hcp coordination than for fcc coordination. However, it is emphasized that the transformation is only temporary and the perfect bcc structure is restored when the dislocation is released from the precipitate.

As shown in the Supplementary Material, coherent bcc precipitates form rather weak obstacles 0.05 ≤ *α* ≤ 0.26. In an effort to rationalize this result, the shear modulus misfit between the bcc W matrix and Re-rich precipitate is assessed. Although the Voigt average of the shear modulus, *G*_*V*_, increases with composition (see Fig. 7b in ref.^19^), the shear modulus in the glide plane, ${\mu }_{\{110\}}^{ < 111 > }$, decreases with composition, both experimentally and by the potential. The values are ${\mu }_{\{110\}}^{ < 111 > }$ = 160, 144 and 118 GPa for *C*_Re_ = 0, 50 and 75 at.% Re, respectively, compared to *G*_*V*_ = 160, 221 and 230 GPa. It is emphasized that a similar decrease is also observed in experiment^29^ and is not an artifact of the used potential^19^. Thus, the observed *τ*_*C*_ cannot be rationalized based on the shear modulus misfit. In fact, based on the shear modulus misfit solely, a bcc precipitate should not form an obstacle for edge dislocation glide.

**Figure 3:**
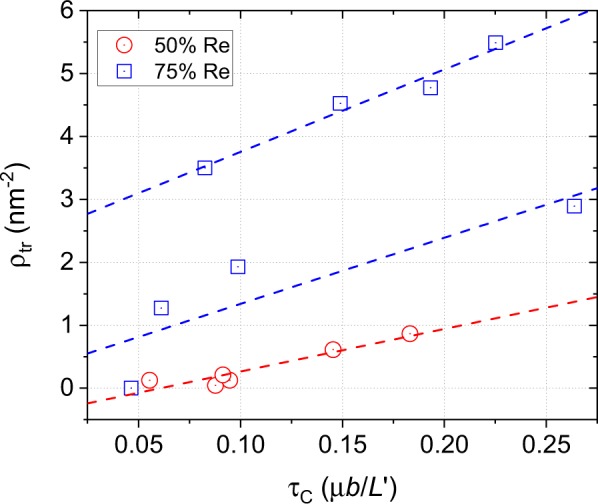
The density of transformed atoms, *ρ*_tr_, as a function of the critical resolved shear stress, *τ*_*C*_, for bcc precipitates containing 50 and 75% Re.

The rationalization of the existence of *τ*_*C*_ can be found in the local and temporary change of coordination of atoms in the precipitate near the dislocation core. To show this, the *ρ*_tr_ versus *τ*_*C*_ is plotted in Fig. 3. The plots show a linear correlation between *ρ*_tr_ and *τ*_*C*_, with *τ*_*C*_ increasing for increasing *ρ*_tr_. Thus, *τ*_*C*_ is controlled by *ρ*_tr_, independent of precipitate shape, *d* and *L*, for a given precipitate composition. It is also observed that *ρ*_tr_ increases with *C*_Re_, which is consistent with the decreasing stability of the bcc phase with *C*_Re_.

It is noted that for precipitates containing 75% Re, two different correlations are found. Between the two, only one goes through the origin. As *τ*_*C*_ is controlled by *ρ*_tr_ and hardening by shear modulus misfit is abscent, the correlation not passing through the origin must be incorrect. The latter correlation is likely the consequence of two competing local minima during the conjugate gradient minimization, leading to two different interaction mechanisms, with one involving more transformed atoms than the other.

### Non-coherent precipitates

In the following, the simulation results for non-coherent precipitates, i.e., *σ*, *χ* and hcp precipitates are presented. All performed simulations for various *C*_Re_, precipitate shape, *d* and *L* are summarized in the Supplementary Material and Methods section. In addition, to further substantiate the transferability of the results, simulations for some key configurations were also performed using the potential by Setyawan *et al*.^30^. As shown in the Supplementary Material, these configurations include a spherical *σ*, a cylindrical *χ* and both spherical and cylindrical hcp precipitates. Except for the interaction with spherical *σ*-precipitates containing 50 and 75% Re, *d* = 3 nm and *L* = 10 nm, all interaction mechanisms are similar for all non-coherent precipitates, regardless of their size, shape, composition or used potential. For the latter two cases, upon contact with the dislocation, the spherical *σ*-precipitates transformed into bcc ones, due to their small size. As such, the observed interaction mechanism is the same as the one previously described.

**Figure 4:**
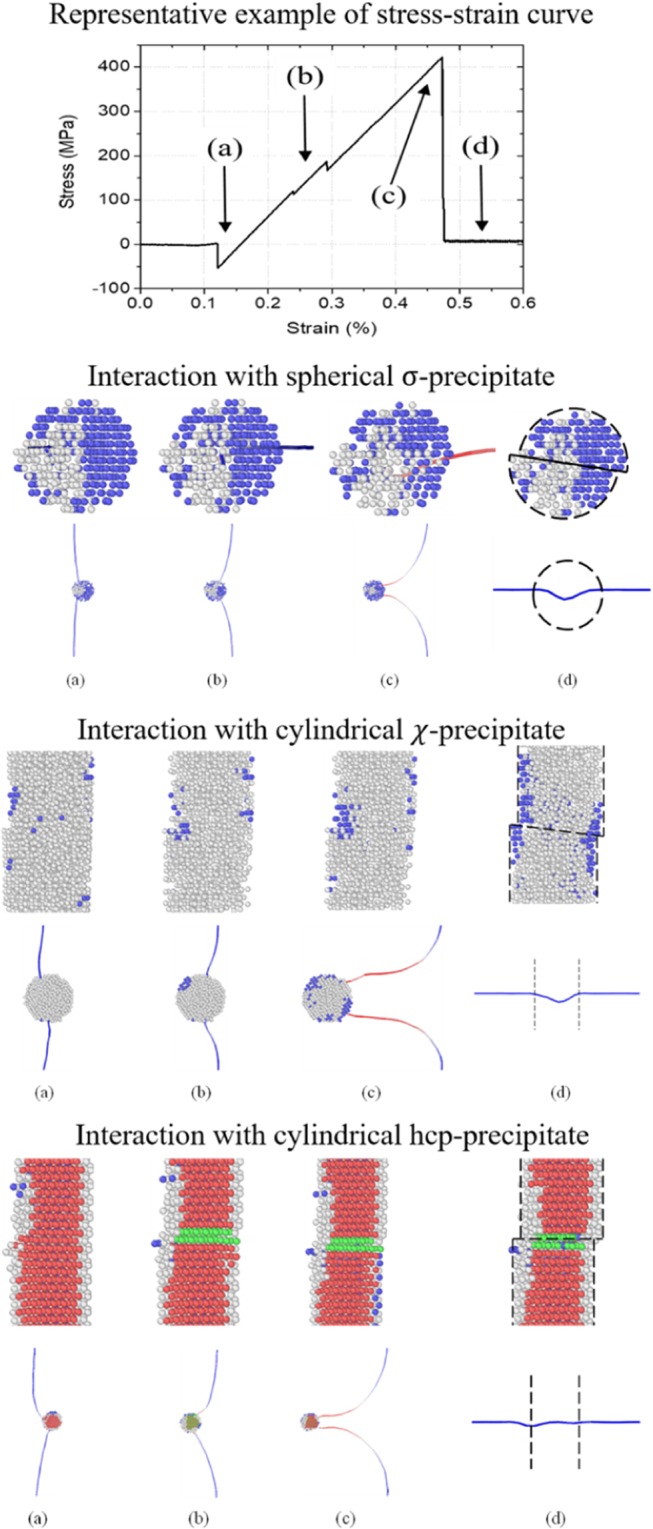
Representative example of a stress-strain curve for the dislocation interaction with a sigma precipitate (similar for chi and hcp precipitates) and the corresponding snapshots. For *σ*, *χ* and hcp precipitates the cases for *C*_Re_ = 50, 75 and 100% Re, *d* = 3, 3 and 2 nm, *L* = 20, 10 and 20 nm are shown. The dislocation line is color coded following its character: blue for pure edge character, red for pure screw character and white for mixed character. Only the Re atoms are visualized and color-coded following a common neighbor analysis: blue – bcc; green – fcc; red – hcp; grey – unidentified, here corresponding to *σ* and *χ*.

A typical stress strain curve for the interaction of an edge dislocation with a non-coherent precipitate is given in Fig. 4. Snapshots corresponding to the different interaction stages of the dislocation-precipitate interaction are provided in the same figure, for *σ*, *χ* and hcp precipitates. For both *σ* and *χ* phases, the interaction with an edge dislocation occurs as follows: a) the dislocation is slightly attracted to the precipitate; b) the dislocation penetrates the precipitate and is pinned by it; c) a screw dipole develops; d) the dipole unpins and leaves a sheared precipitate behind. The screw arms cross-slipped over the precipitate-matrix interface resulting in the formation of a double super jog on the edge dislocation line.

For hcp precipitates, the interaction mechanism is similar to the one for *σ* and *χ* precipitates described above. However, upon penetration of the precipitate by the dislocation, a stacking fault is formed that remains after the dislocation left the (sheared) precipitate. In Fig. 4, the stacking fault is characterized by two layers of atoms with an fcc coordination rather than an hcp one. It is noted that in this case no cross-slip of the screw arms was observed.

**Figure 5:**
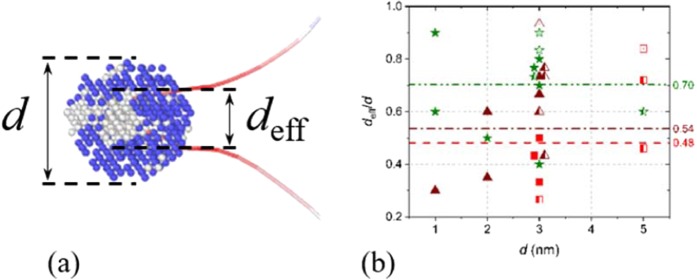
(**a**) Snapshot of a screw dipole drawn from the *σ* precipitate in Fig. 4, to illustrate the difference between *d* and *d*_eff_. (**b**) Plot of *d*_eff_/*d* for all investigated cases. Data points colored in red, brown and green correspond to *σ*, *χ* and hcp precipitates, respectively. Filled and half-open symbols denote cylindrical and spherical precipitates, respectively. The open symbols indicate data obtained using the Setyawan potential. The superposed lines indicate the average *d*_eff_/*d* for spherical *σ*, cylindrical *χ* and all hcp precipitates.

It is observed that even though the *σ*, *χ* and hcp phases are mechanically stable for the investigated compositions (see^19^), part of the precipitate transforms due to the contact with the bcc matrix and interaction with the dislocation. As illustrated in Figs 4 and 5a, the dislocation is pinned by the untransformed part of the precipitate, whose diameter is indicated as the effective diameter, *d*_eff_. As shown in Fig. 5b (and the Supplementary Material), on average, the effective diameter is 50–75% of *d*, with the *σ* phase undergoing the largest transformation and the hcp phase the smallest. Although within the same range, *d*_eff_ obtained using the Setyawan potential seem larger than the other reported values.

## Discussion

**Figure 6:**
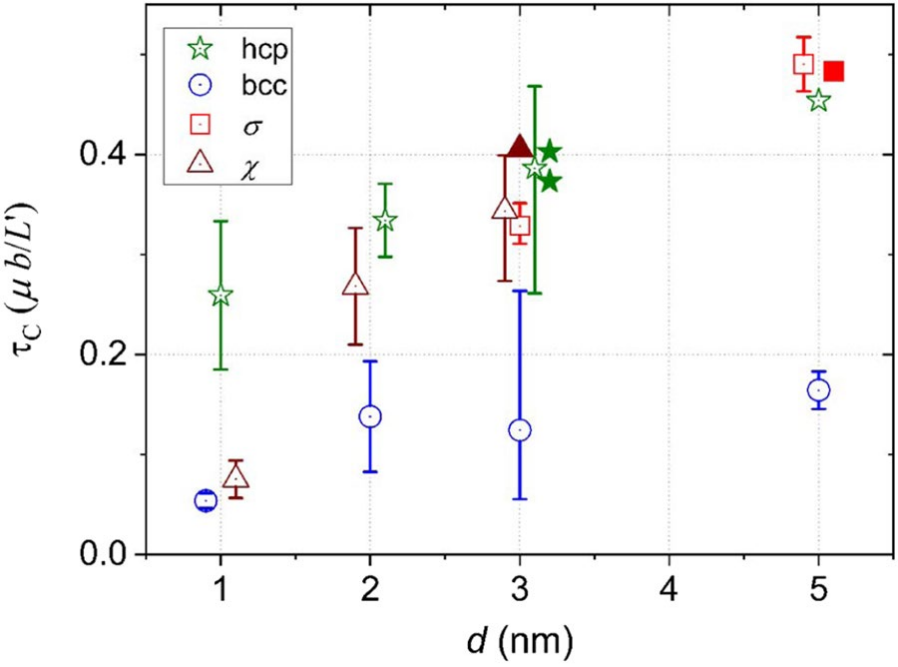
Average critical resolved shear stress resulting from the MS simulations, *τ*_*C*_^MS^, as a function of precipitate diameter, *d*. The average was taken over all simulated precipitate shapes, compositions and *L* for a given phase and *d*. The minimum and maximum values are indicated by the asymmetric error bars. The two cases where the *σ* precipitates transformed into bcc ones were omitted. The filled symbols indicate data obtained from the Setyawan potential.

The computed values for critical resolved shear stress resulting from the MS simulations, *τ*_*C*_^MS^, (reported in the Supplementary Material) vary significantly and no clear trends with precipitate shape, composition or *L* can be identified. In an effort to compare *τ*_*C*_^MS^ for the different phases, *τ*_*C*_^MS^ was averaged over the different shapes, compositions and *L* for each phase. The result is plotted in Fig. 6 as a function *d*. To illustrate the spread in the data, the minimum and maximum values are indicated by the asymmetric error bars.

The figure can be described as follows: *τ*_*C*_^MS^ increases with *d*, and non-coherent precipitates form significantly stronger obstacles than coherent ones for dislocation movement. For the non-coherent precipitates, *τ*_*C*_^MS^ is very similar, as expected given the similar interaction mechanisms. The main differences and large scatter are the result of different fractions of transformed atoms, leading to different *d*_eff_, different *L*_eff_′ = *L* − *d*_eff_ and hence different *τ*_*C*_^MS-eff^ for each interaction. Clearly, the data from the Setyawan potential is in the range of the other data.

In the following, the aim is to describe *τ*_*C*_ for the dislocation interaction with non-coherent precipitates via an analytical model based on the results obtained by the molecular static (MS) simulations. The interaction of an edge dislocation with a non-coherent precipitate is characterized by the appearance of a screw dipole. Therefore, as a first approximation, the theory of Bacon-Kocks-Scattergood (BKS) is applied, which provides the critical resolved shear stress for an impenetrable obstacle as^31^,

1 $${\tau }_{C}^{{\rm{BKS}}}=\frac{\mu b}{2\pi L^{\prime} }[\mathrm{ln}(\frac{1}{{d}^{-1}+{L^{\prime} }^{-1}})+0.7],$$

with *d* and *L*′ given in units of *b*.

**Figure 7:**
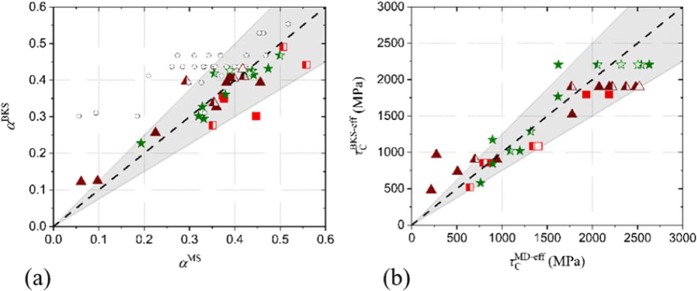
(**a**) Obstacle strength following BKS theory, *α*^BKS^, versus the one from MS, *α*^MS^. The data represented by the empty circles is based on *d* and *L*′, while the other data is based on *d*_eff_ and *L*_eff_′. (**b**) The effective critical resolved shear stress following BKS theory, *τ*_*C*_^BKS−eff^ versus the one from MS, *τ*_*C*_^MS−eff^. Data points colored in red, brown and green correspond to *σ*, *χ* and hcp precipitates, respectively. Filled and half-open symbols denote cylindrical and spherical precipitates, respectively. The shaded area represents a 25% spread around perfect agreement. Open symbols indicate data obtained from the Setyawan potential.

For all investigated cases of non-coherent precipitates, the obstacle strength obtained by BKS theory, *α*^BKS^, is plotted versus *α*^MS^ (the obstacle strength obtained from the MS simulations) in Fig. 7a (data represented by the small open circles). Clearly, there is no correlation between *α*^BKS^ and *α*^MS^; hence application of BKS in the form of Eq. 1 fails to describe the MS simulations.

However, as described in Fig. 5, *d*_eff_ rather than *d* describes the obstacle diameter for the interaction. Therefore, Eq. 1 is applied using *d*_eff_ and *L*_eff_′, leading to ${\alpha }_{{\rm{eff}}}^{{\rm{BKS}}}$ that is compared to ${\alpha }_{{\rm{eff}}}^{{\rm{MS}}}$. The values for ${\alpha }_{{\rm{eff}}}^{{\rm{BKS}}}$ versus ${\alpha }_{{\rm{eff}}}^{{\rm{MS}}}$ are superposed in Fig. 7a. This time, a clear correlation between ${\alpha }_{{\rm{eff}}}^{{\rm{BKS}}}$ and ${\alpha }_{{\rm{eff}}}^{{\rm{MS}}}$ is observed: the BKS theory reproduces most ${\alpha }_{{\rm{eff}}}^{{\rm{MS}}}$ within a 25% spread. Thus, application of BKS for impenetrable obstacles using *d*_eff_ and *L*_eff_′ accurately describes the MS results. As shown in Fig. 7a, the data obtained using the Setyawan potential is consistent with this analysis. This suggests that this analysis is valid beyond the scope of a single potential.

Given the correlations, *d*_eff_ = *Kd*, with *K* a constant depending on the phase as determined in Fig. 5b, the resulting *τ*_*C*_ can be estimated as,

2 $${\tau }_{C}^{{\rm{BKS}}-{\rm{eff}}}=\frac{\mu b}{2\pi (L-Kd)}[\mathrm{ln}(\frac{1}{{(Kd)}^{-1}+{(L-Kd)}^{-1}})+0.7].$$

A direct comparison between the effective critical resolved shear stress from BKS theory, *τ*_*C*_^BKS-eff^ and the effective critical resolved shear stress from the MS simulations, *τ*_*C*_^MS-eff^ is provided in Fig. 7b. Clearly, comparison between theory and MS simulations is satisfactory as most of the MS data is reproduced within a 25% spread. As a result, a simple theory that predicts *τ*_*C*_ for a given phase and precipitate diameter is derived.

Clearly, Eq. 2 also applies to the Setyawan potential. Therefore, although the two potentials show minor differences in *d*_eff_, the resulting model is representative for either potential, thereby strengthening the generality of the results.

To further validate Eq. 2, it is compared to experiments^13,14^, where both precipitate microstructure and radiation hardening is reported. The irradiation hardening was characterized via Vickers micro-hardness tests. By applying a dispersed barrier model, the hardening due to non-coherent precipitates obtained via the Vickers micro-hardness, $\Delta {H}_{V}$, is estimated following^32^,

3 $$\Delta {H}_{V}=6\mu \alpha b\sqrt{Nd},$$

with *N* the precipitate number density. The results following Eq. 3 are compared with experimental data in Table 1. Although the predicted values are within the experimental range, the value for the smallest precipitate size is underestimated. This is likely the consequence of an underestimation of *d*_eff_ by the potential for small precipitates, thereby underestimating its obstacle strength. Thus, *d*_eff_ is likely size-dependent. Nevertheless, the current simplified model presented in Eq. 2 can provide a physically-based measure for the critical resolved shear stress associated to non-coherent *σ* and *χ* precipitates.

**Table 1:** Comparison of the hardening obtained by the Vickers micro-hardness test, $\Delta {{H}}_{V}^{\exp }$, with the one obtained via the BKS theory, $\Delta {H}_{V}^{{\rm{BKS}}}$.

*N* (10^22^/m^3^)	*d* (nm)	${{\boldsymbol{\alpha }}}_{{\bf{eff}}}^{{\bf{BKS}}}({\boldsymbol{\sigma }})$	${{\boldsymbol{\alpha }}}_{{\bf{eff}}}^{{\bf{BKS}}}({\boldsymbol{\chi }})$	${\boldsymbol{\langle }}{{\boldsymbol{\alpha }}}_{{\bf{eff}}}^{{\bf{BKS}}}{\boldsymbol{\rangle }}$	${\boldsymbol{\Delta }}{{\boldsymbol{H}}}_{V}^{{\bf{B}}{\bf{K}}{\bf{S}}}\,({\bf{G}}{\bf{P}}{\bf{a}})$	${\boldsymbol{\Delta }}{{\bf{H}}}_{{\bf{V}}}^{\exp }\,({\bf{G}}{\bf{P}}{\bf{a}})$	Refs
3.9	2.8	0.15	0.17	0.16	0.44	0.9	^13^
41.7	9.5	0.29	0.29	0.29	4.82	4.11	^14^
83.7	6.8	0.24	0.25	0.25	4.89	5.18	^14^

Despite this promising result, a few words of caution and open questions should be stated. It is important to clarify the impact of temperature on the interaction mechanisms and resulting obstacle strength to complement the first results obtained here. The interaction of a dislocation with Re-rich non-coherent precipitates at elevated temperature should account for a possible modification of the precipitate lattice friction, local shifts in the precipitate-matrix interface, as well as local phase transformations, cross-slip and climb of the dislocation arms due to the appearance of a screw dipole, and other thermally activated processes.

It is also important to highlight the general relevance of the present study, which up to now incorporates the investigation of an edge dislocation solely. It is well known that the onset of ductility, i.e., just above the ductile to brittle transition temperature, in bcc materials is controlled by the movement of screw dislocations^33^. The temperature dependence comes from the fact that the thermally activated kink pair nucleation is the critical step in the advance of screw dislocations at low temperatures. However, at elevated temperatures, well above the ductile to brittle threshold, the ductility of polycrystalline bcc metal-based materials is primarily determined by the operation of Frank-Read sources and the subsequent formation/dissolution of dislocation pile-ups (in front of grain boundaries, carbides and other microstructures). Thus, at elevated temperature, the propagation of a screw dislocation is controlled by the movement of kinks and their steady-state concentration.

In this situation, both edge and screw dislocations behave as flexible dislocation lines that can bend around strong obstacles, such that the general models based on the line tension model should apply. Therefore, the presence of the nano-metric precipitates, studied here, should have a similar impact on the motion of both types of dislocations as long as ductile regime is considered. Subsequently, the mechanisms by which the edge dislocation interacts with the non-coherent precipitates observed here should be relevant also for screw dislocations. The validation of this statement requires further investigations involving large scale MD simulations at elevated temperature employing both edge and screw dislocations.

## Conclusions

In this work the interaction of an edge dislocation with coherent and various non-coherent precipitates was studied. Based on the simulations and derived model the following conclusions are reached.

- In the W-Re system, coherent precipitates are weak obstacles. The obstacle strength is controlled by a local and temporary phase transformation from bcc to fcc and hcp. The obstacle strength increases proportionally with the number of transformed atoms.
- Non-coherent precipitates form strong obstacles. The interaction occurs as follows: a) the dislocation is slightly attracted to the precipitate; b) the dislocation penetrates the precipitate and is pinned by it; c) a screw dipole develops; d) the dipole unpins and leaves a sheared precipitate behind. The screw arms cross-slipped over the precipitate-matrix interface resulting in the formation of a double super jog on the edge dislocation line.
- This interaction mechanism for the non-coherent precipitate is independent of the precipitate phase, shape, composition or used potential. Therefore, the interaction mechanism is expected to be valid beyond the scope of the W-Re system.
- Due to its interaction with the dislocation line, non-coherent precipitates are partially transformed. Thereby the effective diameter of the obstacle that pins the dislocation is reduced. This reduction depends on the specific phase of the non-coherent phase. Although different reductions are expected for different systems, this principle is expected to be valid beyond the scope of the W-Re system.
- The obstacle strength of non-coherent precipitates is adequately described by the Bacon-Kocks-Scattergood theory for impenetrable obstacles, provided the effective obstacle diameter is used.
- Application of the model in a dispersed barrier model framework provides a rationalization of the experimentally measured irradiation hardening in the W-Re system.

## Supplementary information

Supplementary information

## Data Availability

The authors declare that all data supporting the findings of this study are available within the paper and upon reasonable request to the authors.
