# Supplementary material for: Assessment of hardening due to non-coherent precipitates in tungsten-rhenium alloys at the atomic scale
Source: Sci Rep. 2019 Nov 7;9:16215. doi: 10.1038/s41598-019-52521-x (PMC6838318; doi:10.1038/s41598-019-52521-x)
Supplement: Supplementary file 1 — Supplementary information [file 41598_2019_52521_MOESM1_ESM.pdf]

Supplementary Information

Assessment of hardening due to non-coherent precipitates in tungsten-rhenium alloys at the atomic scale

G. Bonny*, A. Bakaev, D. Terentyev

SCK•CEN, Nuclear Materials Science Institute, Boeretang 200, B-2400 Mol, Belgium

List of abbreviations

b	– Burgers vector length
BKS	– Bacon-Kocks-Scattergood theory for impenetrable obstacles
d	– Obstacle diameter
d^{eff}	– Effective obstacle diameter
L	– Inter-obstacle spacing
L'	– Free passage length between obstacles
L'_{eff}	– Effective free passage length between obstacles
μ	– Shear modulus of the bcc W matrix
MS	– Molecular Static
$\rho_{\text{tr}}^{\text{max}}$	– Maximum density of transformed atoms
τ_C	– Critical resolved shear stress
τ_C^{eff}	– Effective critical resolved shear stress

Table 1 – Summary of the maximum density of transformed atoms, $\rho_{\text{tr}}^{\text{max}}$, and the critical resolved shear stress, τ_C, for all simulated conditions.

C_{Re} (at.%)	L (nm)	d (nm)	$\rho_{\text{tr}}^{\text{max}}$ (nm^{-2})	τ_C ($\frac{\mu b}{L'}$)
sphere				
50	10	3	0.13	0.09
50	20	3	0.21	0.09
50	20	5	0.61	0.15
50	40	5	0.87	0.18
75	10	3	5.49	0.23
75	20	3	2.89	0.26
cylinder				
50	10	3	0.13	0.06
50	20	3	0.04	0.09
75	10	1	1.27	0.06
75	20	1	0.00	0.05
75	10	2	3.50	0.08
75	20	2	4.77	0.19
75	10	3	1.93	0.10
75	20	3	4.53	0.15

* Corresponding author: gbonny@sckcen.be, giovanni.bonny@gmail.com

Table 2 – Summary of critical and effective critical observed shear stress obtained from MS and the BKS theory for all simulated conditions.

				MS		BKS	
C_{Re} (at.%)	L (nm)	d (nm)	d^{eff} (nm)	τ_C^{MS} $(\frac{\mu b}{L'})$	$\tau_C^{\text{MS-eff}}$ $(\frac{\mu b}{L'_{\mathrm{eff}}})$	τ_C^{BKS} $(\frac{\mu b}{L'})$	$\tau_C^{\mathrm{BKS-eff}}$ $(\frac{\mu b}{L'_{\mathrm{eff}}})$
σ-sphere							
50	10	3	0.0			*	*
50	20	3	0.8	0.31	0.35	0.47	0.28
50	20	5	3.6	0.46	0.51	0.53	0.49
50	40	5	2.3	0.52	0.56	0.55	0.44
75	10	3	0.0			*	*
$ *50*	*20*	*5*	*4.2*	*0.48*	*0.51*	*0.53*	*0.51*
σ-cylinder							
50	10	3	1.0	0.35	0.45	0.44	0.30
50	20	3	1.3	0.34	0.38	0.47	0.35
75	10	3	1.5	0.31	0.37	0.44	0.36
χ-sphere							
50	10	3	1.3	0.28	0.35	0.44	0.34
75	10	3	2.2	0.36	0.40	0.44	0.40
75	20	3	1.8	0.27	0.29	0.47	0.40
100	10	3	2.3	0.38	0.42	0.44	0.41
χ-cylinder							
50	10	3	2.2	0.35	0.39	0.44	0.40
75	10	1	0.3	0.06	0.06	0.30	0.12
75	20	1	0.3	0.09	0.10	0.31	0.12
75	10	2	1.2	0.33	0.36	0.39	0.33
75	20	2	0.7	0.21	0.23	0.41	0.26
75	10	3	2.0	0.40	0.46	0.44	0.39
75	20	3	2.0	0.37	0.39	0.47	0.41
100	10	3	2.0	0.33	0.38	0.44	0.39
$ *75*	*10*	*3*	*2.8*	*0.41*	*0.42*	*0.44*	*0.43*
hcp-sphere							
75	10	3	2.5	0.33	0.35	0.44	0.42
100	10	3	2.7	0.41	0.42	0.44	0.43
100	20	3	2.2	0.43	0.44	0.47	0.43
100	20	5	3.0	0.45	0.50	0.53	0.47
$ *100*	*10*	*3*	*2.7*	*0.37*	*0.39*	*0.44*	*0.43*
hcp-cylinder							
75	10	3	1.2	0.26	0.33	0.44	0.33
100	10	1	0.6	0.19	0.19	0.30	0.23
100	20	1	0.9	0.33	0.33	0.31	0.29
100	10	2	1.0	0.30	0.32	0.39	0.30
100	20	2	1.4	0.37	0.38	0.41	0.36
100	10	3	2.4	0.42	0.44	0.44	0.41
100	20	3	2.3	0.47	0.47	0.47	0.43
$ *100*	*10*	*3*	*2.5*	*0.40*	*0.43*	*0.44*	*0.42*

* Different interaction mechanism, i.e., the same as in the bcc case.

$ Data obtained using the Setyawan potential.
